# High interindividual variability in habitat selection and functional habitat relationships in European nightjars over a period of habitat change

**DOI:** 10.1002/ece3.6331

**Published:** 2020-05-07

**Authors:** Lucy J. Mitchell, Tim Kohler, Piran C. L. White, Kathryn E. Arnold

**Affiliations:** ^1^ Department of Environment and Geography University of York Heslington UK; ^2^ Natural England Humberhead Peatlands NNR Hatfield Moors Doncaster UK

**Keywords:** *Caprimulgus*, functional response, GPS tracking, habitat selection, individual behavior

## Abstract

An animal's choice of foraging habitat reflects its response to environmental cues and is likely to vary among individuals in a population. Analyzing the magnitude of individual habitat selection can indicate how resilient populations may be to anthropogenic habitat change, where individually varying, broadly generalist populations have the potential to adjust their behavior. We collected GPS point data from 39 European nightjars (*Caprimulgus europaeus*) at a UK breeding site where restoration measures have altered large areas of habitat between breeding seasons. We calculated individual habitat selection over four breeding seasons to observe changes that might align with change in habitat. We also analyzed change in home range size in line with change in habitat availability, to examine functional relationships that can represent trade‐offs made by the birds related to performance of the habitat.

Individual explained more of the variation in population habitat selection than year for most habitat types. Individuals differed in the magnitude of their selection for different habitat types, which created a generalist population composed of both generalist and specialist individuals. Selection also changed over time but only significantly for scrub habitat (60% decrease in selection over 4 years). Across the population, individual home range size was 2% smaller where availability of cleared habitat within the home range was greater, but size increased by 2% where the amount of open water was higher, indicating the presence of trade‐offs related to habitat availability. These results highlight that using individual resource selection and specialization measures, in conjunction with functional responses to change, can lead to better understanding of the needs of a population. Pooling specialist and generalist individuals for analysis could hide divergent responses to change and consequently obscure information that could be important in developing effective conservation strategies.

## Graphical abstract

Incorporating individual variation into measures of habitat selection can indicate how resilient populations may be to anthropogenic habitat change. We calculated individual‐level habitat selection and individual functional relationships, over four breeding seasons, to observe changes that might align with change in habitat. Individual variation in habitat selection was high, with both specialist and generalist individuals, whose behavior would have been hidden by using a population mean.

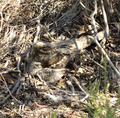

## 1 INTRODUCTION

An animal's decision‐making process should drive it to use habitat that improves its chances of survival and reproductive success (Dussault et al., 2005; Leclerc, 2016; Owen, Swaisgood, & Blumstein, 2017; Roever, Beyer, Chase, & van Aarde, 2014), often making decisions based on one or more environmental cues, such as habitat type or structure. Habitat use, and selection, defined as the strength of use of a habitat compared with its availability (Johnson, 1980; Thomas, Manly, & McDonald, 1992), may reflect quality or configuration, may remain consistent over time (Augé, Chilvers, Moore, & Davis, 2014), or may be functionally responsive to a number of intrinsic and extrinsic factors, such as age, sex, competition, or climate (Godvik et al., 2014; Mauritzen et al., 2003; Treinys, Mozgeris, & Skuja, 2016). Functional responses refer to a change in movement behavior or habitat use in response to a change in habitat availability and may represent ecological trade‐offs related to habitat type (Mabille, Dussault, Ouellet, & Laurian, 2012), conspecific interaction and competition (Buskirk & Millspaugh, 2006; Jones, 2001; Lesmerises, Déry, Johnson, & St‐Laurent, 2018), avoidance of predators (Mao et al., 2005), or human influence (Karelus, McCown, Scheick, van de Kerk, & Oli, 2016; Sawyer, Nielson, Lindzey, & McDonald, 2006).

Analyzing functional responses is important in terms of understanding behavioral flexibility (Godvik et al., 2014; Leclerc et al., 2016; Lesmerises et al., 2018), the costs and benefits of different habitats, and the ability of a species to respond to spatial and temporal landscape change (Boggie, Collins, Donnelly, & Carleton, 2018; Lesmerises et al., 2018; Mauritzen et al., 2003). Functional responses might also indicate the presence of an ecological trap, where an animal is responding to a cue in the environment that has become decoupled from the actual processes at play (Berger‐Tal et al., 2011; Dussault, Pinard, Ouellet, Courtois, & Fortin, 2012). For example, Baxter, Baxter, Dahlgren, and Larsen (2017) found that Greater Sage‐Grouse (*Centrocercus urophasianus*) showed a positive functional response to landscape change by strongly selecting new, mechanically cleared habitats, compared with other existing land‐use types, but this then produced much lower breeding success, a phenomenon echoed by Demeyrier, Lambrechts, Perret, and Grégoire (2016). A recent study found that Moose (*Alces alces*) habitat selection changed with both habitat availability and home range size, indicating a direct response to the absolute amount of particular habitat types (Ofstad et al., 2019).

### 1.1 Individual contribution to population‐level habitat selection

Habitat preferences and responses to change may not be consistent within populations. The direction and magnitude of behavioral responses may vary among individuals and be repeated within individuals (i.e., individual specialization; Bolnick et al., 2003; Forsman & Wennersten, 2016; Nussey, Wilson, & Brommer, 2007). Populations can contain individuals that display both generalist and specialist tendencies (Patrick & Weimerskirch, 2017; Phillips, Lewis, González‐Solís, & Daunt, 2017), where generalists possess a broader niche and exploit a wider range of resources than specialists, whose diet or habitat choice is narrower and often more rigid (Roughgarden, 1974; Wilson & Yoshimura, 1994). A high degree of specialization should encourage higher efficiency in the foraging individual (Garnick, Di Stefano, Elgar, & Coulson, 2016). However, `this can also mean these individuals are potentially less able to switch to a different set of resources and can therefore be more sensitive to change (Polito et al., 2015; Wilson & Yoshimura, 1994). The benefits of specialization are more numerous when resources are abundant and individuals are able to segregate their resource use from conspecifics (Maldonado, Bozinovic, Newsome, & Sabat, 2017). However, where animals are utilizing ephemeral prey and stochastic resources in a heterogeneous environment (Patrick & Weimerskirch, 2017), generalist individuals that use multiple resource types may be more opportunistic and thus have a better chance of maintaining individual condition and passing on their traits to their offspring (Wilson & Yoshimura, 1994).

Quantifying individual variability and how it drives population responses (Nussey et al., 2007) can identify subpopulations in need of extra protection, or those individuals that may “buffer” a population when faced with large‐scale resource change (Forsman & Wennersten, 2016; Phillips et al., 2017). However, the population‐level implications of changing habitat selection in response to availability, such as altered fitness and reproductive success (Phillips et al., 2017), or survival of adults and young (Benson, Mahoney, & Patterson, 2015; Dussault et al., 2012; Losier et al., 2015), are not well‐understood (Mason & Fortin, 2017). Linking behavioral responses in resource selection to demographic consequences is needed in order to create appropriate management or protection interventions, to ensure species’ continued survival (Germain & Arcese, 2014; Roever et al., 2014). Individual variation can be incorporated into habitat selection studies through comparison of habitat use and availability within each individual's home range (i.e., “third‐order” selection; Johnson, Nielsen, Merrill, McDonald, & Boyce, 2006). Quantifying habitat selection at this level can reveal responses to change that may be hidden by pooling individuals (Leclerc et al., 2016; Lesmerises & St‐Laurent, 2017). This can provide an insight into population variation, including consistency in foraging decisions among and between individuals (Leclerc et al., 2016), and differences driven by biological variation between sexes (Ofstad et al., 2019), as well as population dynamics (Baxter et al., 2017; Losier et al., 2015) that can aid future management (Allen & Singh, 2016; Tanner et al., 2016).

### 1.2 Study rationale

In 2014, more than €4 million of European Union LIFE + funding was acquired for the restoration of the Humberhead Peatlands National Nature Reserve (NNR), South Yorkshire, designated as a Special Protection Area (SPA) in 2000, for its population of breeding European nightjar (Joint Nature Conservation Committee, 2010, 2015; Natural England, 2013). The Humberhead Peatlands NNR is separated into two sites: Thorne Moor (Lat: 53.636, Lon: −0.898) and Hatfield Moor (Lat: 53.545, Lon: −0.938), together totaling 2,887 hectares. Despite long‐term peat extraction, the NNR remains the largest area of this habitat type in the UK (Joint Nature Conservation Committee, 2015).

The restoration project aimed to increase wet bog habitat to improve peatland stability and improve the diversity of peatland plant and invertebrate species through mechanical and hand removal of birch woodland and damming of drainage channels. Concurrently, the funding aimed to increase the breeding population of nightjars on the site by 15%, by improving the open habitat available to them in which they could breed; however, the potential impacts of this habitat manipulation on the population were not fully known. Thus, we aimed to investigate the behavioral responses of the breeding nightjar population to the substantial compositional and structural change presented here.

**FIGURE 1:**
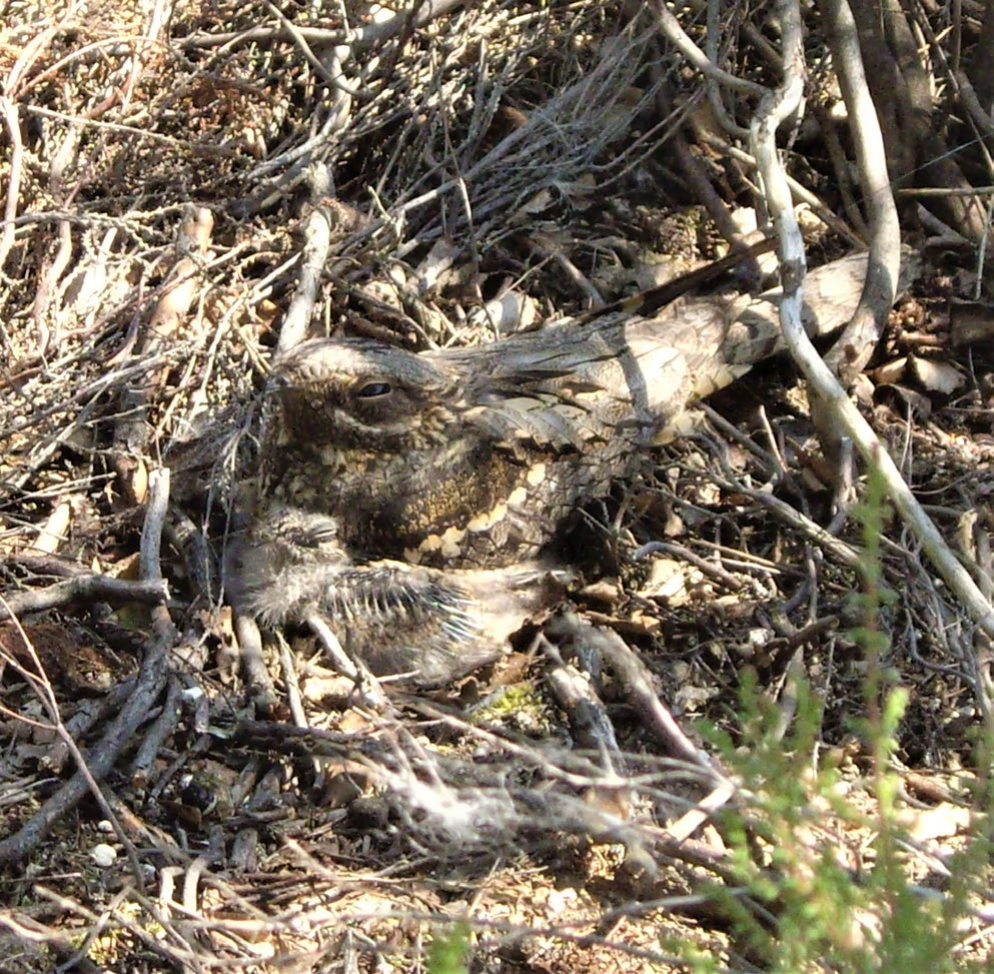
Adult female nightjar brooding one juvenile. Taken on Thorne Moor, South Yorkshire, June 2018 by Lucy Mitchell

The European nightjar is a breeding migrant to the UK and is a bird typically of dry heathland and woodland sites (Figure 1; Berry, 1979; Bright, Langston, & Bierman, 2007; Cramp, 1985). As a result of its habitat requirements, namely the need for dry, well‐draining ground on which to lay their two eggs, the species is sporadically distributed nationally, meaning that there is limited information on their foraging behavior and specific habitat preferences (Evens, Beenaerts, Witters, & Artois, 2017; Sharps, 2013; Verstraeten, Baeten, & Verheyen, 2011; Wichmann, 2004). Birds will fly between 1 km (Palmer, 2002) and 7 km (Evens et al., 2017) from their nest sites to locate more favorable or less competitive habitat, which needs to be rich in their main food source of moths and beetles, for which they forage both on the wing and from a perch (Cramp, 1985; Sharps, 2015). Recent radio‐ and GPS‐tracking studies of nightjars in the UK, Belgium, and Spain show that the use of coniferous plantation including clear‐felled coupes, as well as grazed grassland, heathland, and birch scrub, is common (Alexander & Cresswell, 1989; Conway et al., 2007; Morris, Burges, Fuller, Evans, & Smith, 1994; Sharps et al., 2015). Work by Camacho, Palacios, Sáez, Sánchez, and Potti (2014) and Evens et al. (2017), Evens et al. (2018) showed that nightjars used complementary “functional” habitats for segregated breeding and foraging, highlighting the importance of maintaining a mosaic of habitats in a configuration that reduces the distance between these areas (Camacho et al., 2014; Evens et al., 2018). As the nightjar is a relatively range‐limited species, detailed information on individual habitat selection and foraging movements is needed to measure behavioral, and potentially functional, responses to planned habitat change.

### 1.3 Study aims

In order to understand how a habitat‐specialist, insectivorous bird responds to large‐scale landscape alteration, we obtained fine‐scale data on individual home range size and habitat selection. We quantified nightjar home range and habitat selection behavior over the four‐year period of landscape change, by analyzing GPS point data, which provided us with useful land management information on a suitable scale.

We asked whether nightjar home ranges and habitat selection ratios varied between sexes and/or individuals. We hypothesized that regardless of year, differing parental roles would create a significant difference in home range size but not habitat selection, between males and females. We anticipated clear trends towards selection of “typical” nightjar habitats such as heather and woodland, both when observing home range habitat availability and within‐range selection (Sharps et al., 2015), across all years, by all birds, with a low level of individual variation.

With regard to change over time, we asked whether nightjar home ranges and habitat selection would change over the 4 years of management works, as a result of local‐ and landscape‐level habitat changes. We hypothesized that both composition of nightjar home ranges and within‐home range habitat selection for woodland, heather, and scrub habitats would increase over the study period. We hypothesized that this would be in response to a decrease in birch woodland and scrub, and an increase in cleared habitat.

Related to this, we asked whether we would be able to see a functional response by the nightjars to the changed availability in habitat. We hypothesized that home range size would increase over the course of the restoration works and that the mechanism behind both an increase in home range size and habitat selection strength, as mentioned above, would relate to the decrease in invertebrate‐rich foraging resources within nightjar territories. This would cause the birds to forage over a wider area, due to areas having been cleared, but to be more selective within this area, because key foraging resources have been removed or divided. We also hypothesized that increased wetland habitat types would also push nightjars to forage over wider areas.

## 2 MATERIALS AND METHODS

### 2.1 GPS point data collection

GPS point data were collected during nightjar breeding seasons (June to August) in 2015–2018 (Hatfield Moor) and 2016–2018 (Thorne Moor), via miniature, archival GPS tags (Pathtrack). Birds were captured using 12‐ and 18‐m‐long, 40‐mm hole mist nets (Ecotone), with the addition of a tape lure playing male breeding calls. Mist netting, ringing, tagging, and use of a breeding season tape lure were all done under license from the British Trust for Ornithology (BTO), and all underwent ethical review at the University of York. We aimed to capture equal numbers of birds from the two Moors and within all major habitat types in order not to bias our results. Birds, once captured, were ringed, measured, and weighed, to ensure they were of sufficient weight to carry a tag (for ethical reasons, we did not exceed 3% of the bird's bodyweight). Obtained GPS point data were used to estimate home range size, movement, and habitat selection of adult birds of both sexes. Fixes were collected every 3 min (2015–2016) or 5 min (2017–2018) from 21:00 to 05:00 and were accurate to ±30 m (Pathtrack Company information and our own stationary tests). In total, 45 tracking devices provided between 2 and 16 nights of data from 41 individual birds. One tracking device failed after two nights and one device was accidentally retrieved after three nights, so these were excluded from the analyses. Additionally, two birds dispersed midway through the season, giving unreasonably large home ranges over the timescale analyzed. Thus, data from 41 tags (*n* = 39 birds) were subsequently processed and analyzed (2015: *n* = 4; 2016: *n* = 11; 2017: *n* = 14; 2018: *n* = 14). In 2018, three birds were tagged and successfully retrieved for a second time, permitting a direct interannual comparison of home range size and habitat selection; all other birds were only caught during 1 year. Data were cleaned and transformed into trajectory format using packages adehabitatHR (Calenge, 2006) and maptools (Bivand & Lewin‐Koh, 2013) to allow production of home range contour shapefiles and habitat selection metrics in R (v. 3.5.1.).

### 2.2 Habitat mapping

To estimate habitat availability and selection within each bird's home range and how this changed between years, high‐resolution unmanned aerial vehicle (UAV) photography of both Thorne and Hatfield Moors was acquired in April 2016. This was translated into a high‐resolution (5 × 5 m) habitat map, sufficient for the purposes of identifying nightjar habitat selection, given the resolution of the GPS point data. Classification of the habitat map combined unsupervised image classification in ArcMap (v. 10.4.1) and manual mapping of areas using a handheld GPS unit. Fourteen habitat categories were selected (Appendix S1) and an arbitrary value was attributed to all values outside of the NNR boundary to represent “off‐site” areas, which were comprised of a mixture of arable farming, industry, and residential areas, including allotments and gardens. We updated the map annually before the breeding season, once new areas of scrub clearance had been completed, allowing us to observe use and any change in use, in specific ages of cleared habitat from year to year (i.e., brand‐new clearance, plus 1 or 2 years of vegetation regrowth) (see Appendix S2 for maps displaying the annual habitat change).

### 2.3 Home range modeling

Home ranges were created as spatial polygons in R (using package adehabitatHS; Calenge, 2006), using the movement‐based kernel density estimation method (MKDE; Benhamou, 2011), to estimate individual home ranges and therefore habitat availability and consequent use. The MKDE treats a collection of consecutive GPS point data as an autocorrelated trajectory and therefore is more appropriate for a highly mobile bird. The MKDE contrasts with other commonly used methods (e.g., minimum convex polygons (MCPs) and kernel density estimators (KDEs); Boyce, Vernier, Nielsen, & Schmiegelow, 2002), by modeling occurrence data (Fleming & Calabrese, 2017) and by accounting for nonindependence between points. The difficulty in defining the true availability of different habitat types to individuals highlights the importance of the choice of estimator (Mitchell, White, & Arnold, 2019; Stark, 2017) and the importance of including movement behavior in the process of home range calculation (Benhamou & Cornélis, 2010; Martin, Calenge, Quenette, & Allainé, 2008; Van Moorter, Rolandsen, Basille, & Gaillard, 2015). The use of the MKDE better characterizes the movements of a “goal‐oriented” animal searching a landscape (Calenge, Dray, & Royer‐Carenzi, 2009). As MKDEs place weight on the area between points rather than the points themselves, this allows distinct areas to be connected by regular use of common corridors, particularly relevant for birds that frequently commute to a feeding area, as nightjars have been shown to do (Camacho et al., 2014; Evens et al., 2018). Using the MKDE, tracking duration is influential in both home range and habitat selection calculation, and as a result, birds were compared and collectively analyzed over a six‐day period, in line with the minimum achieved by all tags in the dataset. This allowed the data to be standardized for reasonable comparison (Mitchell et al., 2019). Once home ranges had been calculated, we compared home range sizes between sexes and years using linear regression, where home range size was the dependent variable, and sex and year were independent factors. We then extracted the habitat within the polygons to quantify habitat composition of individual home ranges, before continuing to calculate habitat selection.

### 2.4 Habitat selection

Home range‐level habitat selection, also known as third‐order selection (Johnson et al., 2006), compares available habitat (the percentage, %, of a habitat type available within a home range) with used habitat (designated by the GPS fix locations) within the boundaries of an individual's home range. Habitat selection ratios (Manly, McDonald, Thomas, McDonald, & Erickson, 2002) were created using adehabitatHS (Calenge, 2011). These ratios provide a value that is proportional to the probability of use of an area or habitat type and represents a type of resource selection function (RSF; Manly et al., 2002) more commonly used when there is only one dependent variable (here, we have used one variable—habitat type— summarized in 14 categories; Appendix S1). A selection ratio with a value >1 indicates the use of a habitat away from random (i.e., the habitat is being selected for; larger values suggest higher selection strength), whereas a value <1 represents habitat avoidance. Significant selection for or against a particular habitat, across the population of tracked individuals, can be identified when 95% confidence intervals do not overlap one.

To understand better the spread of habitat selection behavior across the population, we also calculated two measures of individual specialization, primarily used for dietary specialization studies (following Fodrie et al., 2015, and Navarro et al., 2017). Firstly, we calculated Monte Carlo resampled Araujo's *E* (*E_obs_)*, which runs on a continuum from 0, where individuals are generalist, that is, overlapping in their resource selection, to 1, where individuals are specialized, that is, nonoverlapping (Araujo, Layman, & Bolnick, 2011; Zaccarelli, Mancinelli, & Bolnick, 2013). Secondly, we calculated Monte Carlo resampled version Roughgarden's proportional measure of within‐individual components to total niche width (WIC/TNW), used to describe the partitioning of variation in resource use within the population (Roughgarden, 1979; Zaccarelli et al., 2013). Roughgarden's measure uses a Shannon–Weaver formula for discrete count data according to the number of units of each resource type used by each individual (Zaccarelli et al., 2013). In this instance, when the value of WIC/TNW approaches 0 individuals are more specialized, using only subsets of the population resources, and when it approaches 1, individuals are more generalized, using the full population niche. Both measures were Monte Carlo resampled on 999 iterations within package “RInSp” (Zaccarelli et al., 2013), to provide a null value against which to compare the calculated value. Both measures allow us to understand whether individual variation in resource use is strong or weak among all individuals or mixed within the population (i.e., there are both generalist and specialist individuals present).

### 2.5 Changes in habitat selection between years

To test for any difference in habitat selection between years and sexes, potentially as a result of large‐scale habitat change, we used linear mixed models, where selection ratio was the dependent variable, and year and sex were entered as fixed factors, with individual as a random factor, to account for noise created by divergent individual selection behaviors (Bates, Mächler, Bolker, & Walker, 2015). For the three birds tracked in two separate years, we wanted to quantify differences in the selection ratios of these birds to understand whether there was consistency in their behavior. We quantified selection ratios in both years and tested for significant differences between years using a linear model, where selection ratio was the dependent variable.

### 2.6 Changes in home range size with habitat availability

In order to understand the functional relationships between nightjar home range size and habitat availability (i.e., percentage of each habitat type present within the home range), single habitats were grouped into functional categories, according to their structure, vegetation type, and level of moisture. The categories were woodland, scrub, open and dry (heather and bracken), cleared (previously temporally defined clearance categories pooled into one), wetland (cotton grass and wetland), and water.

To test for differences in home range size between sex, year, and availability of different habitat types, we used linear models in R (similar to Ofstad et al., 2019), where home range size (ha) was the dependent variable, and all habitat terms and year were used as fixed effects. Lack of data (i.e., only one value per individual home range) meant we could not use a random effect structure here. We included all noncorrelated habitat categories within the model as a starting, global model (availability of woodland, cleared and wetland habitat, and open water). We sought to find the best model using single‐term deletions and AIC comparison in package MuMin (Barton, 2015). We judged model fit using the Akaike weight and comparison of AIC values between models.

## 3 RESULTS

### 3.1 Nightjar home range size and associated variation

Nightjar 95% home ranges varied between sexes and years, although not quite significant (*F*_4,38_:2.67, *p* = .05, *R* ^2^: .14; Table S2a). Mean male home range size was smaller at 74.36 ha (±*SD* 87.78), whereas mean female home range size was 131.11 ha (±*SD* 119.96), but large confidence intervals for the coefficients of the linear model, and large standard deviations on mean values, showed there was strong individual variation (see Table S2b).

### 3.2 Changes in habitat availability and within‐home range habitat selection

Site management for restoration concentrated on removal or mature woodland and reduction in efficiency of drainage channels by introducing dams to the site. Birch woodland removal produced substantial changes in landscape composition and configuration, influencing the availability of woodland and cleared habitat across the whole site, and within nightjar home ranges over time (Figures S1 and S2). Woodland decreased at a site level but not significantly (Kruskal–Wallis: 2.89, *df* = 3, *p* = .41). However, it was significantly less available within nightjar home ranges (regardless of home range level) in 2017 by 22% (*β*: −12.73 (95% CI: −23.6 to −1.85); mean woodland availability in nightjar home ranges in 2015:31.5%, mean 2017:19%) and in 2018 by 23% (*β*: −12.47 (−23.3 to −1.62); mean 2018:18.1%) compared with 2015. In contrast, cleared habitat became more available on site, by between 400% and 900% (Figure S2b), but a lack of data points meant this was not significant (K‐W: 2.46, *df* = 3, *p* = .48). Within nightjar home ranges, cleared habitat increased significantly from an average of 0.25% in 2015 all other years (mean 2016:9.6%; *β*: 1.63 (0.3–2.93), 2017:13.7%; *β*: 2.08 (0.8–3.37); 2018:12%; *β*: 1.69 (0.45–2.93); Table 1; Table S4). While wetland habitat did increase within home ranges after 2015 (from an average of 1% to >12% in 2016 and >17% in 2017; Table S4), the increase was not significant (Table 1), nor was it significant at an overall site level (K‐W: 0.82, *df* = 3, *p* = .85; Figure S2d).

**TABLE 1:** Coefficients and 95% confidence intervals from linear models used to test for differences in availability of different habitat types (%) within nightjar home ranges between year and level (core/wider) of home range

Parameter	Woodland		(log) Scrub		Open, dry		(log) Clearance		(log) Wetland	
	*β*	(95% CI)	*β*	(95% CI)	*Β*	(95% CI)	*β*	(95% CI)	*β*	(95% CI)
Intercept	31.46	(21.21 to 41.71)	1.52	(0.82 to 2.21)	27.69	(9.58 to 45.79)	0.17	(−0.95 to 1.29)	0.44	(−0.9 to 1.78)
Year 2016	−6.67	(−17.62 to 4.27)	−0.34	(−1.14 to 0.47)	1.21	(−19.12 to 21.21)	***1.63***	***(0.30 to 2.93)***	1.2	(−0.21 to 2.62)
2017	***−12.73***	***(−23.6 to −1.85)***	0.03	(−0.75 to 0.81)	−3.42	(−22.93 to 15.95)	***2.08***	***(0.80 to 3.37)***	1.4	(−0.02 to 2.81)
2018	***−12.47***	***(−23.3 to −1.62)***	0.25	(−0.52 to 1.02)	4.16	(−15.19 to 23.35)	***1.69***	***(0.45 to 2.93)***	1.34	(−0.07 to 2.75)
Individual variation	10.17	(8.27 to 12.9)	0.55	(0.00 to 0.79)	17.84	(12.85 to 22.82)	0.92	(0.00 to 1.32)	1.33	(1.08 to 1.69)
Residual variation	0.93	(0.49 to 2.78)	0.41	(0.21 to 0.81)	2.48	(1.17 to 11.03)	0.63	(0.28 to 1.3)	0.06	(0.03 to 0.2)

Note

Significant results (where 95% confidence intervals do not overlap 1) are highlighted in bold.

**FIGURE 2:**
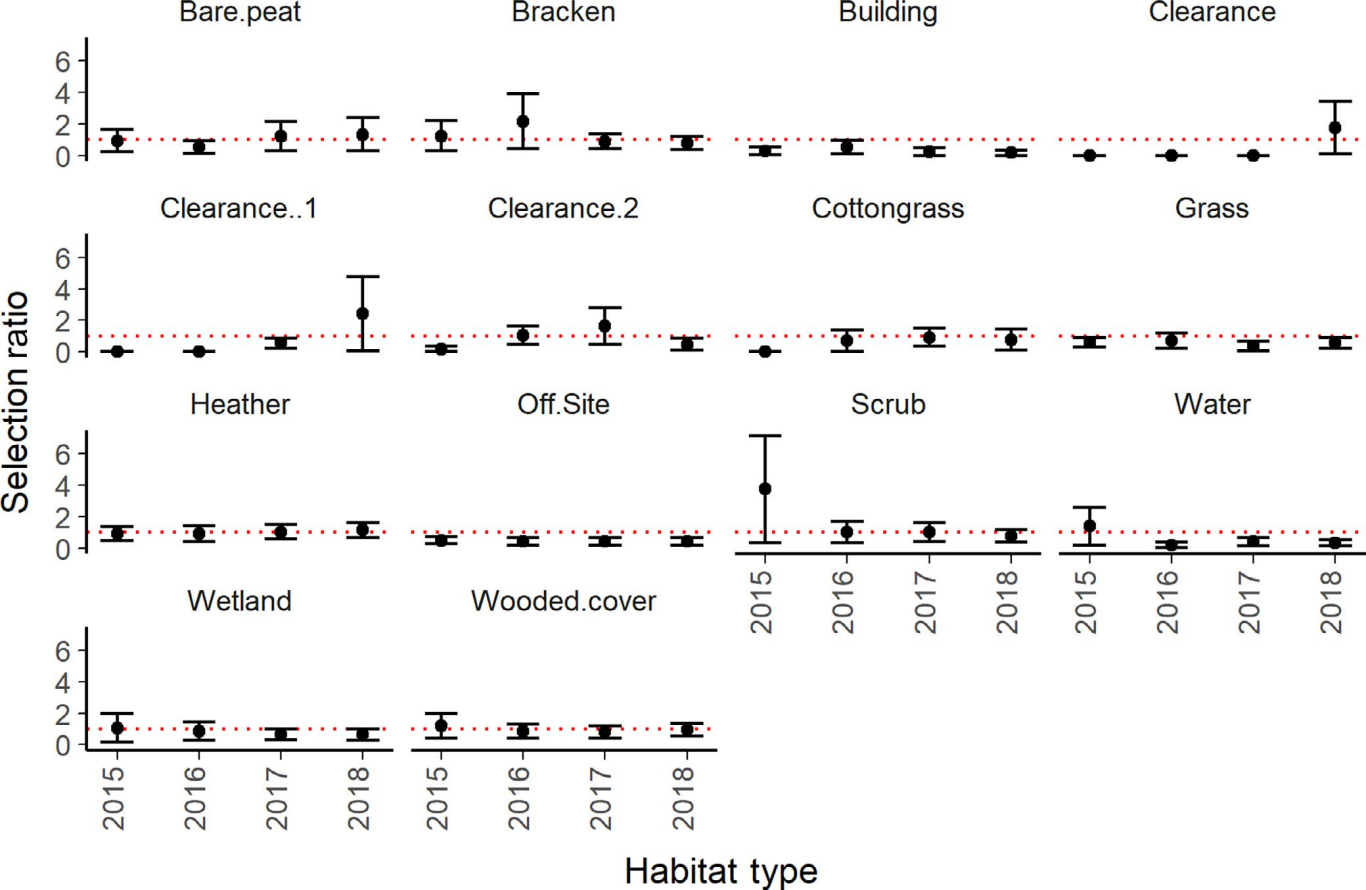
Mean habitat selection ratios for individual nightjars (*n* = 43) within 95% home range on Hatfield and Thorne Moor 2015–2018. Data plotted on a square root scale for visibility. Habitats are separated to allow between‐year comparison. Boxes represent interquartile ranges, and whiskers extend to 1.5 × IQR. Red dashed line at 1 represents line of selection (above = selected for; below = avoided)

Average selection ratios for the nightjar population across 4 years of study varied strongly and indicated individual differences within the population (Table 2, Figure 2). No single habitat type was used to the same extent by all individuals in the population, but multiple birds used heather (mean ± *SE*: 1.07, ±0.13), bracken (1.79 ± 0.81), and woodland (0.82 ± 0.11), as well as cleared habitat (2015/16:2.01 ± 1.79; 2016/17:1.24 ± 0.47; 2017/18:0.98 ± 0.91). Mean habitat selection values for these four habitat types were close to 1, indicating use in line with availability, over more than one breeding season, although large standard errors indicated that the range of individual values was wide (Figure 2). In particular, there were consistently strong selection behaviors by several individuals for cotton grass‐dominated habitat (highest value of 6.1; mean ± *SE*: 0.59 ± 0.21), which directly contrasted strong avoidance by other individuals. Several birds used multiple habitat types evenly, resulting in a lack of individual selection (Figure 2, Table 2). Although selection for different habitat types varied, there was more consistency in the habitat types avoided. Grass‐dominated areas, including agricultural areas off‐site, and open water were all significantly avoided by all birds (mean grass selection ratio (±*SE*): 0.3 ± 0.08; mean off‐site: 0.2 ± 0.03; mean water: 0.28 ± 0.14).

**TABLE 2:** Coefficients and 95% profile‐computed confidence intervals from mixed linear models testing for a significant difference in selection ratios for each habitat type between years within nightjar home ranges

Parameter	Scrub		Woodland		Heather		Bare peat		Clearance + 1	
	*β*	(95% CI)	*β*	(95% CI)	*β*	(95% CI)	*β*	(95% CI)	*β*	(95% CI)
Intercept	2	(1.28 to 2.72)	0.87	(0.51 to 1.23)		(0.19 to 1.08)	0.58	(−0.17 to 1.33)	−0.2	(−1.01 to 0.6)
Year 2016	**−1.39**	(−2.12 to −0.64)	−0.32	(−0.7 –0.05)		(−0.52 to 0.42)	−0.06	(−0.77 to 0.65)	−0.33	(−1.11 to 0.49)
Year 2017	**−1.21**	(−1.9 to −0.51)	−0.34	(−0.71 to 0.02)		(−0.4 to 0.5)	−0.1	(−0.81 to 0.61)	0.55	(−0.22 to 1.32)
Year 2018	**−1.12**	(−1.82 to −0.42)	−0.19	(−0.5 5 to 0.18)		(−0.29 to 0.62)	−0.05	(−0.76 to 0.65)	0.55	(−0.21 to 1.31)
Sex—male	−0.4	(−0.83 to 0.03)	−0.11	(−0.32 to 0.11)		(−0.32 to 0.21)	−0.07	(−0.52 to 0.38)	0.27	(−0.21 to 0.76)
Individual variation	0.62	(0.37 to 0.67)	0	(0.00 to 0.25)		(0.00 to 0.42)	0.66	(0.45 to 0.76)	0.71	(0.39 to 0.91)
Residual variation	0.14	(0.24 to 0.39)	0.32	(0.22 to 0.4)		(0.18 to 0.49)	0.03	(0.16 to 0.26)	0.07	(0.03 to 0.54)
Marginal *R* ^2^	0.3		0.12				0.004		0.24	
Conditional *R* ^2^	0.96		0.12				0.99		0.99	

Parameter	Wetland		Bracken		Cotton grass		Clearance + 2	
	*β*	(95% CI)	*β*	(95% CI)	*β*	(95% CI)	*β*	(95% CI)
Intercept	0.59	(0.14 to 1.05)	0.57	(−0.12 to 1.25)	−0.03	(−0.58 to 0.46)	−0.18	(−0.86 to 0.51)
Year 2016	−0.22	(−0.72 to 0.27)	0.28	(−0.42 to 0.98)	0.06	(−0.39 to 0.66)	0.52	(−0.14 to 1.18)
Year 2017	−0.32	(−0.77 to 0.14)	−0.22	(−0.9 to 0.45)	0.13	(−0.23 to 0.83)	0.62	(−0.04 to 1.4)
Year 2018	−0.16	(−0.65 to 0.33)	−0.28	(−0.95 to 0.39)	0.13	(−0.21 to 0.81)	0.4	(−0.42 to 1.06)
Sex—male	−0.05	(−0.32 to 0.23)	0.24	(−0.18 to 0.65)	0.03	(−0.23 to 0.39)	0.28	(−0.13 to 0.69)
Individual variation	0.37	(0.00 to 0.51)	0.57	(0.00 to 0.75)	0.19	(0.31 to 0.57)	0.6	(0.00 to 0.77)
Residual variation	0.14	(0.12 to 0.46)	0.22	(0.11 to 0.66)	0.07	(0.07 to 0.34)	0.1	(0.05 to 0.6)
Marginal *R* ^2^	0.05		0.16		0.05		0.11	
Conditional *R* ^2^	0.88		0.89		0.9		0.98	

Note

Individual bird was included as a random effect. Significant results (where 95% confidence intervals do not overlap 1) are highlighted in bold. Marginal/conditional *R* ^2^ values = amount of variation explained by fixed effects/fixed and random effects.

Habitat selection of all habitat types did not differ significantly between males and females (Table 2), but individual variation explained a large proportion of the overall habitat selection variation (*R* ^2^ values, Table 2). Marginal *R* ^2^ values, that is, the amount of variation explained by the fixed effects (year and sex), were generally very low (.05–.3). In contrast, condition *R* ^2^ values were mostly above .88, with the exception of woodland and heather selection ratios (condition *R* ^2^ of .12 and .3, respectively).

Measures of individual specialization gave similar results and indicated a moderately specialized population, where although among‐individual variation was lower than within‐individual variation, overlap between individuals was not great enough to class the population as wholly generalist (Fodrie et al., 2015). Roughgarden's total niche width (TNW: 2.08) was mostly comprised of within‐individual components (WIC: 1.18) as opposed to between‐individual components (BIC: 0.9). This gave a *WIC/TNW* ratio of 0.57 (*p* = .001), significantly less generalist than expected (Table 3, Figure S3). Araujo's MC‐resampled *E_obs_* also indicated a population with significantly more specialized individuals than expected, when compared against the null model (*E_obs_* = 0.65, range of null *E* values: 0.06–0.08 (*p* = 0) on a continuum of 0–1, 1 being a specialist population; Table 3; Figure S3).

**TABLE 3:** Results from two measures of individual specialization

Roughgarden's total niche width				
WIC	BIC	TNW	WIC/TNW	*p* value
1.18	0.9	2.08	0.57	.001

Araujo's MC resampled *E*		
*E*_obs_	Null *E*	*p* (*E*_sim_ => *E*_obs_)
0.65	0.07	0

Note

Roughgarden's measures of the within‐ and between‐individual components (WIC/BIC) of a population niche width and the total niche width (TNW); WIC/TNW is the ratio of the within‐individual components to the total niche width; *p* value = significance of the ratio; followed by Araujo's *E*, the null *E* calculated from simulated data, and the p value representing a significant departure of the from the expected *E* value.

### 3.3 Between‐year variation including within‐individual comparison

Despite the significant alteration of woodland habitat, selection for woodland by nightjars did not change significantly over time (mean woodland selection ratio (±*SE*) 2015:1.41 (±0.62); 2016:0.71 (±0.2); 2017:0.67 (±0.16); 2018:0.91 (±0.15); Table 2, Figure 2). Change in selection ratios over time was only significant in all years for scrub, selection for which decreased linearly by over 60% after 2015 (mean scrub selection ratio (±*SE*) 2015:14 (±11.5); 2016:1.04 (±0.47); 2017:1.03 (±0.35); 2018:0.61 (±0.14); Table 1; Table S4), despite no reduction in this habitat type. In contrast, selection ratios for new, one‐year‐old, and two‐year‐old clearance increased over time, but this was not significant due to high individual variation (Table 2).

Although among‐individual variation was high, three birds tracked during two separate breeding seasons displayed remarkably consistent home range placement and habitat selection between years, with individual home range overlap varying from 61% to 78%. There was no significant difference seen between any selection ratios of two of the three birds (linear regression, nj1; *F*_1,46_:0.008, *p* = .93; nj2; *F*_1,46_:0.15, *p* = .70). For nj3, a significant change in selection between years was identified (*F*_1,46_:5.44, *p* = .02). Specifically, nj3 increased its use of woodland of 10% and decreased its use of bracken‐dominated habitat by 1%, which was enough to produce a significant change in selection ratio, given the availability of these habitat types.

### 3.4 Functional responses to habitat availability

**FIGURE 3:**
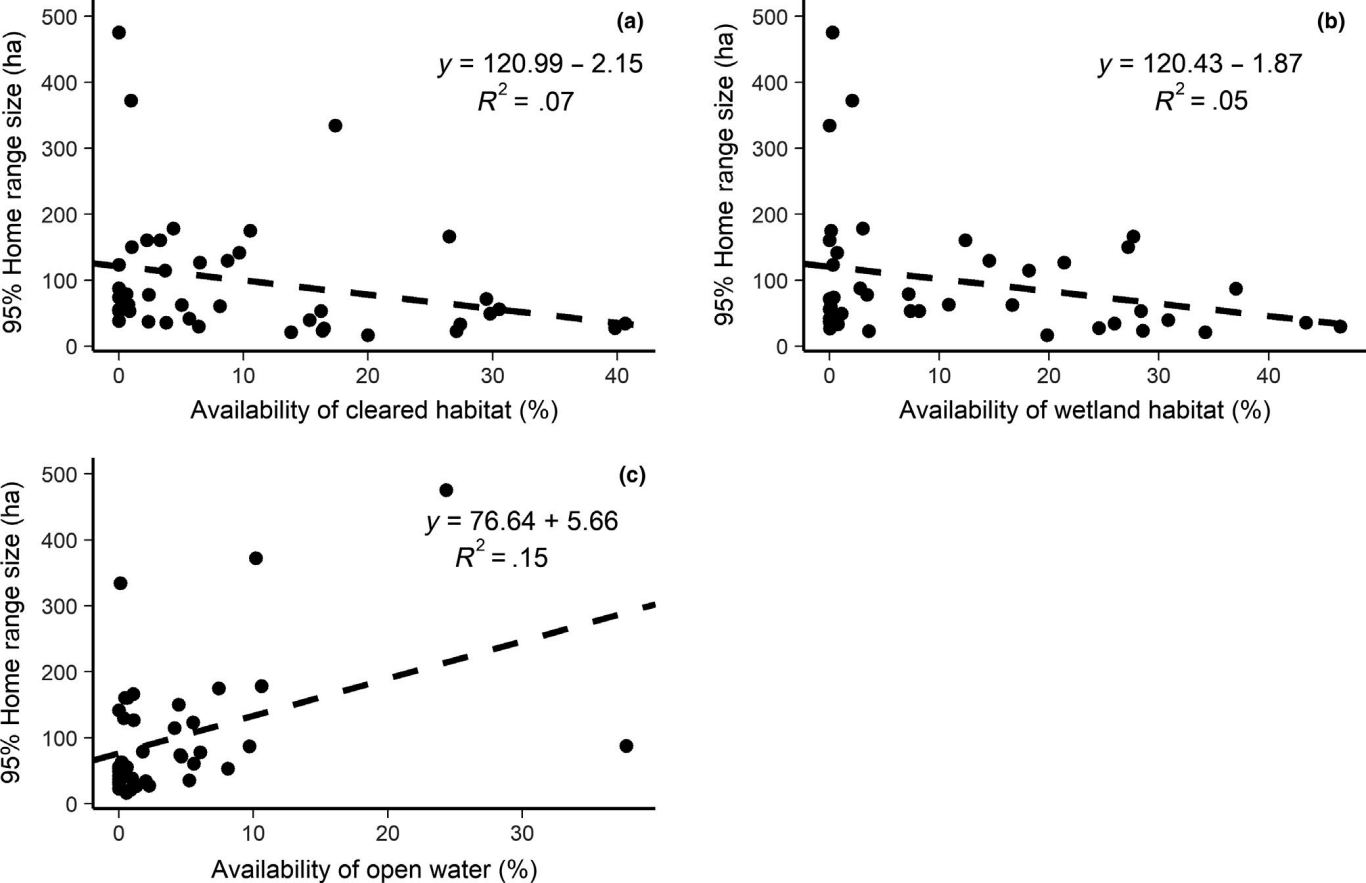
Linear relationship between (a) home range size (hectares) and availability of cleared habitat (%);(b) home range size (hectares) and availability of open water (%). All linear models run with 95% home range size

Four linear models containing a number of habitat types along with year were within delta 2 AICc of each other and were therefore model‐averaged (Table 4). Across the population, nightjars showed functional responses to habitat availability, but this was only significant when specifically talking about availability of cleared habitat (Table 5). Home range size decreased by 1.98% when available cleared habitat increased by 1%. Although not significant, home range size also decreased by 1.1% when wetland habitat increased by 1%. There was additionally a weak increase in home range size of 2.02% when there was an increase of 1% open water (Figure 3, Table 5).

**TABLE 4:** Model selection table for linear models testing for functional responses in nightjar home range size according to availability of different habitat types

Model selection table	AICc	*df*	Delta	Weight
Intercept + Cleared % + Water % + Wetland %	**99.1**	**5**	**0**	**0.35**
Intercept + Cleared % + Wetland % + Year	**99.8**	**7**	**0.65**	**0.25**
Intercept + Cleared % + Water %	**100.4**	**4**	**1.32**	**0.18**
Intercept + Cleared % + Water % + Wetland % + Year	**100.5**	**8**	**1.4**	**0.17**
Intercept + Cleared % + Water % + Wetland % + Woodland % + Year	103.5	9	4.44	0.04

Note

Highlighted models are all within delta 2 of each other, so have been model‐averaged (values presented in Table 5). Delta: difference in AICc between two models; weight: Akaike weight.

**TABLE 5:** Coefficients and 95% confidence intervals from the best linear model (by AIC and Akaike weight; Table 4) used to test for functional responses in 95% nightjar home range size to habitat availability and over time

Home range size (hectares)		95% CI	
Variable	*β*	Lower	Upper
Intercept	4.451	3.888	5.015
Availability (%) of open water	**0.021**	**−0.005**	**0.063**
Availability (%) of cleared habitat	−0.02	−0.04	−0.0002
Availability (%) of wetland habitat	−0.011	−0.03	0.002
Year 2016	0.071	−0.685	1.005
Year 2017	−0.095	−1.088	0.664
Year 2018	0.217	−0.339	1.315

Note

*R* ^2^: .295.

*F*_6,36_: 3.925; *p* = .004.

Coefficients and confidence intervals for models of functional responses in the 95% home range are model‐averaged estimates for all models within Δ 2 of top model. Significant results are highlighted in bold.

## 4 DISCUSSION

### 4.1 Nightjar home range variation

Nightjar home ranges were smaller for males than for females, and smaller in 2017 than other years, but none of these differences were significant because of overlap in confidence intervals owing to high individual variation. Strong individual variation was also found in Mitchell et al. (2019) where individuals were responsible for more variation in size than year, week, and parameters such as the number of days over which the bird was tracked. High individual variation in home range is common in other species (Börger et al., 2006; Patrick & Weimerskirch, 2017); therefore, using the unit of the individual, rather than generalizing across the population, is clearly important.

### 4.2 Nightjar habitat selection and variation over time

Overall, there was a weak, nonsignificant population‐level preference for drier habitats, which agrees with previous findings (Alexander & Cresswell, 1989; Jenks, Green, & Cross, 2014; Sharps et al., 2015). However, several individuals selected strongly for wetland habitat, which was unexpected and contributed to the overall lack of significant population‐level habitat selection. The use of atypical habitats, such as cotton grass‐dominated “bog,” was also found by Evens et al. (2017). There was also a strong contrast between birds that did and did not use cleared habitat; nightjars nesting in manipulated areas selected for this habitat intensely, but those that did not nest there, did not use it at all, even for foraging visits.

Despite a decrease in woodland habitat onsite, selection for this habitat did not significantly change, which was contrary to our predictions. In fact, woodland and heather habitat were the most stable in terms of selection (i.e., ratios did not increase or decrease significantly over time) and were also less influenced by individual variation. The greater stability in selection for these habitats does indicate use by multiple individuals, but elsewhere, the dichotomy of responses to different habitats among individuals highlights the importance of considering the full breadth of behaviors, not just the average.

There are several mechanisms that may be contributing to the diversity of behaviors. Firstly, preference for open areas that have been provided by new clearance work has been reported previously (Sharps et al., 2015; Verstraeten et al., 2011; Wichmann, 2004). These areas provide desirable nesting habitat as well as foraging habitat, and this multifunctionality potentially explains strong use by birds in this study. Secondly, the results related to the availability and selection of woodland perhaps imply that there is a threshold amount of woodland needed for foraging within their home range (Angelstam, Bütler, Lazdinis, Mikusinski, & Roberge, 2003; Zielewska‐Büttner, Heurich, Müller, & Braunisch, 2018), which is met by most of the individuals here resulting in a lack of selection for this habitat. Thirdly, the use of a wide variety of habitat types demonstrates this species’ ability to be flexible according to the distribution of food resources, which may not be habitat‐specific, but may change according to short‐term weather conditions as well as habitat structure, which influences the ease of prey capture (Sharps et al., 2015).

Individual variation was particularly strong; variation attributed to both fixed and individual effects was often three times that for the fixed effects alone. This variation suggested that the population as a whole was generalist in its use of habitats but that there were both specialist (strong selection for one or two habitat types) and more generalist (weak selection for multiple habitat types) individuals present. This assertion was supported by the total niche width and Araujo's *E* values, and is also consistent with other studies (Kotler & Brown, 1988; Wilson & Yoshimura, 1994). Individuals partially overlapped in their resource use, using separate, unique sets of resources otherwise.

Although generalist behavior can result in costs to individuals (Patrick & Weimerskirch, 2017), where species need to respond to stochastic variation in prey resources, generalist behavior should be more prevalent (Wilson & Yoshimura, 1994). For insectivorous species such as nightjars, a generalist diet could support them through seasonal and annual fluctuations in invertebrate populations. If generalist diets are adopted by the majority of individuals, then differences between individuals in habitat selection may not be due to habitat‐specialist diets, but may relate more to spatial segregation due to competition. Birds of different age or body condition may possess higher foraging efficiency in particular habitats (Patrick & Weimerskirch, 2017). Indeed, Kotler and Brown (1988) state that mosaics of habitat, such as that created at this nightjar breeding site, can lead to different strengths of habitat selection and coexistence of multiple phenotypes.

### 4.3 Within‐individual comparison

Intraindividual consistency in home range placement and habitat selection was demonstrated in three birds. Despite being based on only three individuals, interannual consistency is common in site‐faithful long‐distance migrants, particularly where individuals have been successful in breeding in previous seasons (Patrick & Weimerskirch, 2017; Wakefield et al., 2015). Often seemingly generalist populations can contain specialist individuals who display remarkable consistency from year to year (Phillips et al., 2017); however, we require data from more individuals on this timescale to produce a robust estimate of the level of consistency within this population.

### 4.4 Functional responses in home range size

Functional responses were apparent for birds within several habitat types, across the whole population. Home range size changed as a result of the availability of cleared and open water habitats. This suggests that there are foraging‐related constraints affecting all individuals to some extent, which ultimately determine the utility of an area and should be taken into account when managing for this species. Contrary to our hypotheses, where home ranges contained more cleared habitat, home range was smaller. Habitat management can result in direct habitat loss, but can also create “novel” areas of habitat that attract animals by changing the physical structure and availability of food resources (Hodson, Fortin, & Bélanger, 2010; Summerville, Bonte, & Fox, 2007; Summerville & Crist, 2002). Birch woodland has a high invertebrate diversity (Webb, Clarke, & Nicholas, 1984), particularly of beetles and moths, the preferred prey of nightjars (Sharps, 2013). This should mean that birds with access to these areas do not have to travel as far to find foraging resources. However, the clearance of large areas of woodland can make it easier for nightjars to obtain prey since it provides increased edge and open habitats, allowing them to forage more in these areas of regrowing vegetation rather than in dense, mature birch woodland. In contrast, the increase in home range size where the amount of open water was higher indicated a lack of suitability of these areas for foraging. We believe this was related to both the abundance of prey and the specific taxa present that are more likely to be chironomids and diptera that are more commonly preyed upon by bats (Rydell, Entwistle, & Racey, 1996).

Other influences on habitat selection such as density dependence and conspecific interactions, perhaps related to age, or body size (Delaney & Warner, 2017), have not been investigated here, due to a lack of robust data. The interaction between an individual's phenotype and the environment in which it persists might consequently impact the evolution and heritability of traits (Alonzo, 2015), such as Great tits (*Parus major*) that used different subhabitats according to their particular morphology (Gustafsson, 1988); collecting detailed morphological data from captured individuals may be able to illuminate this. Individual variation such as that in this population of nightjars is important for the flexibility of the population in the context of future habitat change. Populations with varying phenotypes should contain at least some individuals that can succeed (Forsman & Wennersten, 2016) and pass on traits to their offspring. Land managers can foster multiple phenotypes by providing different habitat types can bolster a population also coping with environmental change.

## 5 CONCLUSIONS

We have provided evidence of the need to include individual‐level information on habitat selection alongside population functional responses (Leclerc et al., 2016) to fully appreciate the use of a site and the value of its resources by a population. Pooling disparate individuals to calculate a mean habitat selection value can hide substantial individual variation present in a population, which can have significant consequences for ecological processes (Schirmer, Herde, Eccard, & Dammhahn, 2019). Nightjar habitat selection values varied significantly among individuals. Divergent habitat selection among individuals demonstrated different specialist behaviors alongside more generalist individuals, in which little selection was evident. This combination contributed to apparent overall population flexibility, where individual specialization was tempered by some common habitat use by all individuals. By providing a range of habitat types, configurations, and growth stages, thus increasing the opportunities available for both specialist and generalist foragers, land management can promote varying phenotypes, potentially leading to greater population resilience to environmental stressors. For a previously declining species, reliant on a threatened prey group, the use of multiple, different habitat types should be a positive indicator for its survival in the future.

## CONFLICT OF INTEREST

The authors declare no conflicts of interest.

## AUTHOR CONTRIBUTIONS

**Lucy J. Mitchell:** Conceptualization (lead); data curation (lead); formal analysis (lead); investigation (lead); methodology (lead); validation (equal); writing – original draft (lead); writing – review & editing (lead). **Tim Kohler:** Supervision (supporting); writing – review & editing (supporting). **Piran C. L. White:** Conceptualization (equal); supervision (equal); validation (equal); writing – original draft (supporting); writing – review & editing (supporting). **Kathryn E. Arnold:** Conceptualization (equal); funding acquisition (lead); supervision (equal); writing – original draft (supporting); writing – review & editing (supporting). 

## Supporting information

Appendix S1‐S3

## Data Availability

All GPS data used for analysis in the publication are available within the NERC EIDC data repository: https://doi.org/10.5285/d5cc1b92‐6862‐4475‐8aa1‐5936786d12ab (Mitchell, Kohler, White, & Arnold, 2020).
