# Supplementary material for: High interindividual variability in habitat selection and functional habitat relationships in European nightjars over a period of habitat change
Source: Ecol Evol. 2020 May 7;10(12):5932–45. doi: 10.1002/ece3.6331 (PMC7319154; doi:10.1002/ece3.6331)

Appendix S1

Table S1: Vegetation categories defined during the habitat map creation process and their associated number coded into the raster file.

| **Number** | **Vegetation type** |
| --- | --- |
| 1 | Open water – significant pools; do not dry out in summer. |
| 2 | Bare peat – unvegetated peat; sometimes pooled with water overwinter. |
| 3 | Wooded cover – primarily birch trees of >2 metres tall. Covers mature scrub, as well as woodland. |
| 4 | Wetland vegetation – a combination of species that can tolerate high groundwater level, including *Juncus* spp., *Eriophorum* spp., sedges, willow and some birch. |
| 5 | Cottongrass – *Eriophorum* dominated areas – often bordering open pools or on deeper, wetter peat. Contains large amounts of *Sphagnum* – perhaps closest to ‘true bog’. |
| 6 | Bracken – drier areas including ‘baulks’, and some cleared areas, dominated by bracken. |
| 7 | Heather – areas representing more ‘wet heath’ type habitat with heather of varying age and condition. |
| 8 | Scrub – areas covered by young, re-growing birch scrub, often with little other ground vegetation. |
| 9 | Clearance (2016/17) – Recent (<1 year) clearance. Low levels of ground vegetation, still newly lying brash and/or chippings. |
| 10 | Clearance +1 (2015/16) – Older clearance; often becoming dominated by bracken and rhododendron regrowth depending on thickness of brash layer. |
| 11 | Grass – Often external to the NNR, but also present on Lindholme Island. Managed low level grass areas. |
| 12 | Building – Only present on Hatfield due to tarmacked roads through to peat works. |
| 13 | Off Site – covers all areas not included within the boundary of the NNR including agricultural land, urban development and old industry. |
| 14 | Clearance (2017/18) – Newly cleared habitat, only present on Hatfield in 2018. |

Appendix S2

Figure S1: Map displaying areas of habitat change (2015-2018) through clearance of woodland, on a) Thorne Moors and b) Hatfield Moors. Unmanaged habitat refers to heather, woodland, scrub, peatland and wetland areas that have not had any direct management, either through mechanical or hand-clearance during 2015 to 2018.


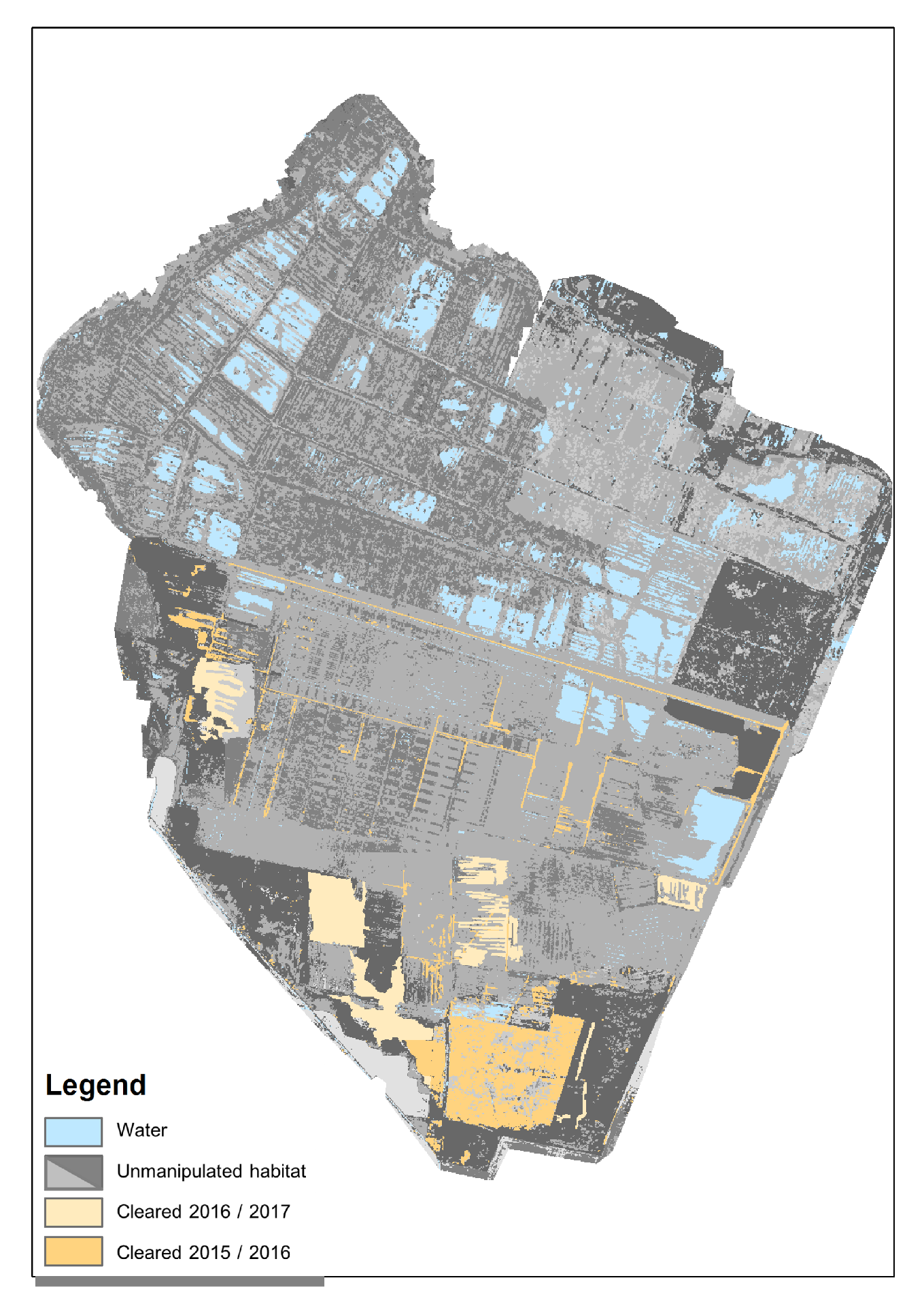


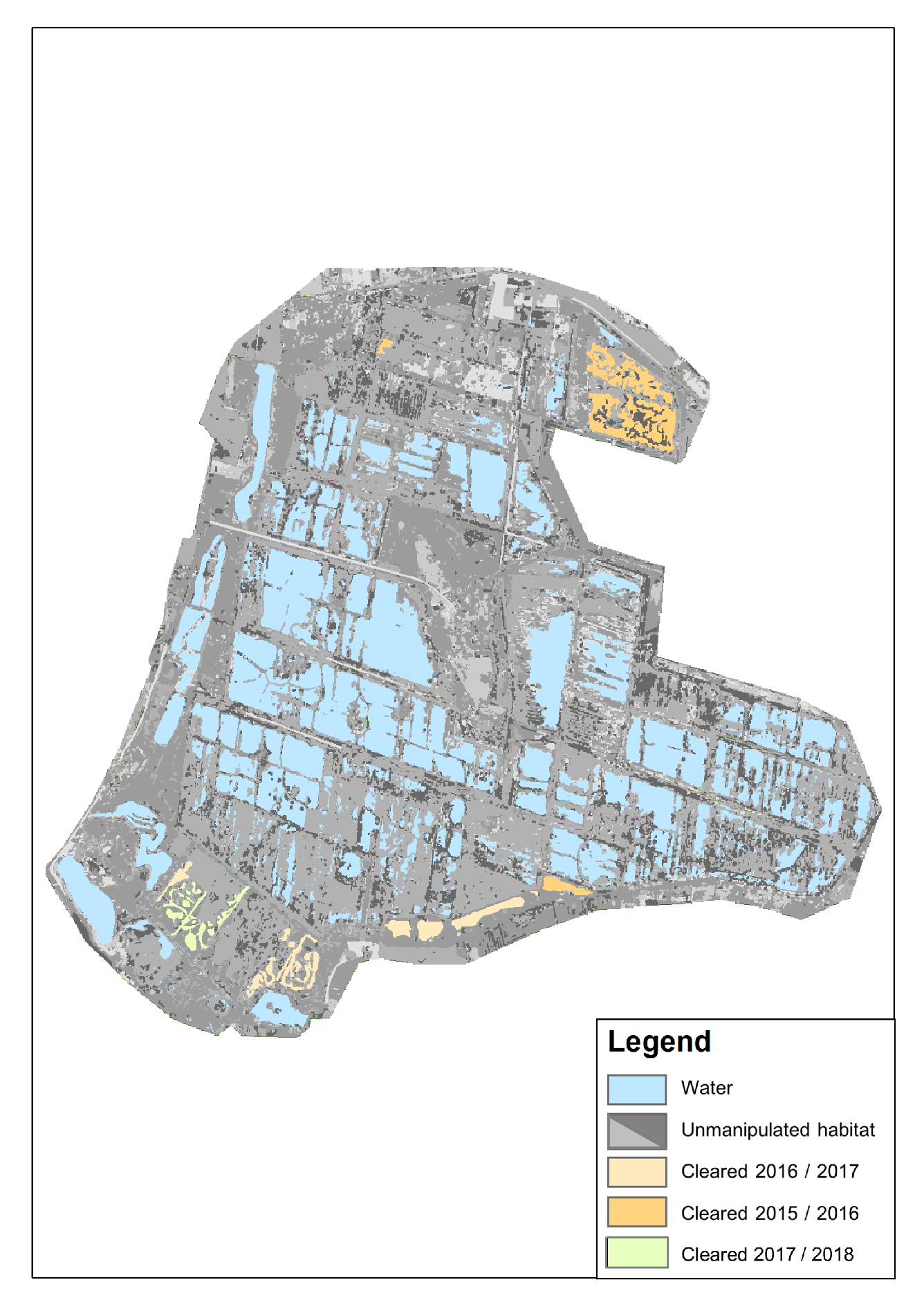


Figure S2: Change in availability (in hectares) of a) woodland, b) cleared habitat, c) heather habitat, and d) wetland habitat on Hatfield and Thorne from 2015 – 2018.


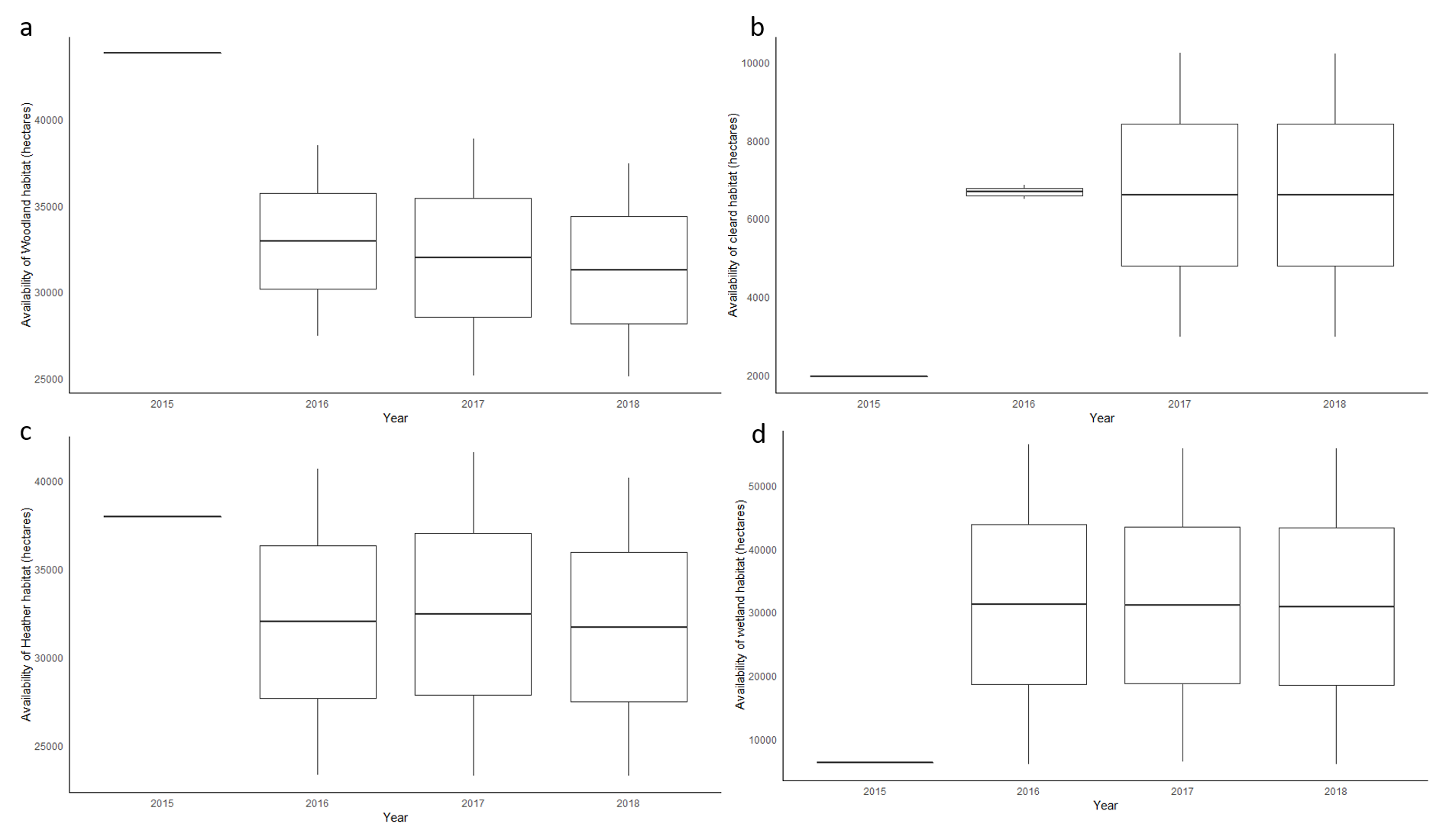


Appendix S3

Table S2a): Mean home range sizes (hectares) with standard errors, for all birds GPS tagged (n = 43) from 2015 to 2018 on Thorne and Hatfield Moors, South Yorkshire, UK; A2b: Coefficients and 95% confidence intervals from the model testing for significant differences in home range size between years and sexes.

a)

b)

Table S3: Mean % of habitat types available within home ranges of all GPS-tagged nightjars (n = 43) from 2015 to 2018 on Thorne and Hatfield Moors, South Yorkshire.

Table S4: Mean habitat selection ratios for all birds GPS tagged (n = 41) for all habitat types (n = 14) from 2015 to 2018 on Thorne and Hatfield Moors, South Yorkshire, UK. Values <1 = habitat type avoided; values ~1 = habitat type used in line with availability; values >1 = habitat selected for.

Figure S3: Distribution plots for Monte-Carlo simulated data for a) WIC/TNW (Roughgarden, 1979) and b) Araujo’s E (Araujo *et al.*, 2011) produced using package ‘*RInSp*’ in R (Zaccarelli *et al*., 2013). Blue line displays the actual mean WIC/TNW or E value for the sample; density bar and 95% confidence intervals represent simulated data results.


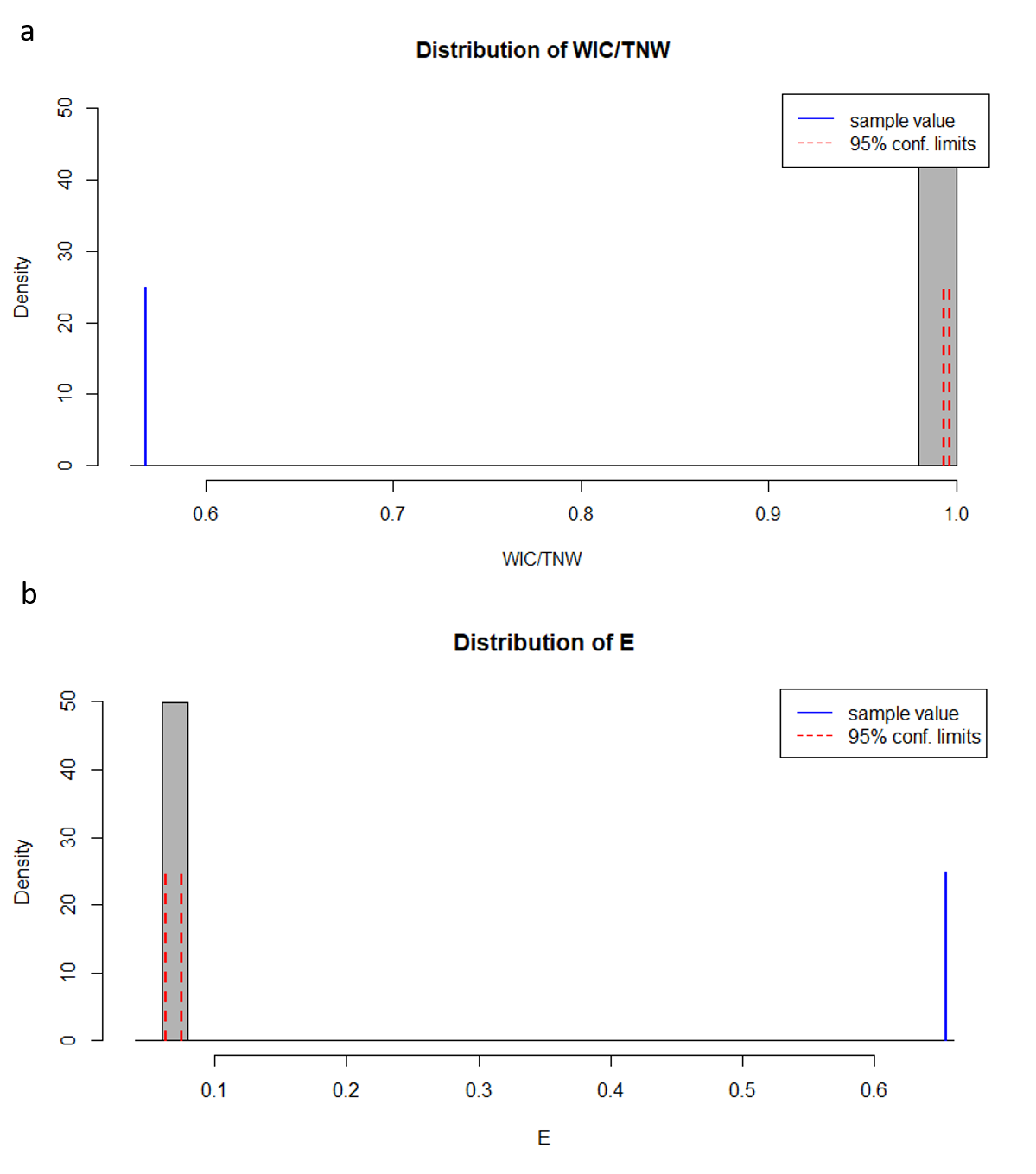

Supplement: Supplementary file 1 — Appendix S1‐S3 [file ECE3-10-5932-s001.docx]
